# Supercritical Fluid‐Processed Multifunctional Hybrid Decellularized Extracellular Matrix with Chitosan Hydrogel for Improving Photoaged Dermis Microenvironment

**DOI:** 10.1002/adhm.202403213

**Published:** 2025-03-20

**Authors:** Seol‐Ha Jeong, Jae Jun Kang, Ki‐Myo Kim, Mi hyun lee, Misun Cha, Su Hee Kim, Ji‐Ung Park

**Affiliations:** ^1^ Department of Plastic and Reconstructive Surgery Seoul National University Boramae Hospital Seoul National University College of Medicine Seoul 07061 Republic of Korea; ^2^ Bio‐max Institute Seoul National University Seoul 08826 Republic of Korea; ^3^ R&D Center Medifab Co. Ltd 5 Gasan digital 1‐ro, Geumcheon‐gu Seoul 08594 Republic of Korea

**Keywords:** adipose dECM, carboxymethylchitosan hydrogel, decellularization, dermal fillers, heart dECM, photoaging, supercritical fluid processing

## Abstract

To address the demand for reconstructive procedures in extensive subcutaneous tissue defects and significant dermis matrix loss, vascularized adipose tissue regeneration is essential for maintaining volume after material degradation. Accordingly, a double‐crosslinked hydrogel that combines polyethylene glycol (PEG)‐crosslinked carboxymethyl chitosan (CMC) with a hybrid decellularized extracellular matrix (dECM) is developed. The dECM, sourced from porcine adipose and cardiac tissues, processed using a supercritical fluid technique (scCO_2_‐EtOH) retains 1.5–5‐fold more angiogenic and adipogenic cytokines than that processed using traditional methods. This hybrid dECM‐based filler demonstrates excellent physical properties and injectability, with injection forces being significantly less than that for crosslinked hyaluronic acid (HA) fillers. Upon incubation at 37 °C, the storage modulus of the fillers increases substantially, eventually enhancing their moldability from additional crosslinking and the thermosensitive nature of collagen. Assessments in a UVB‐induced photoaging mouse model indicate that the material maintains superior shape stability, durability, and supports vascularized tissue regeneration, reduces inflammation, and enhances VEGF expression and ECM maturation more effectively compared with that using other fillers. These promising results suggest that the material can serve as a highly effective multifunctional solution for injectable regenerative medical applications and is well‐suited for potential clinical trials.

## Graphical abstract

These findings advance tissue repair by offering a multifunctional filler that improves large‐scale volume retention, elasticity, and support for vascularized tissue growth. The decellularized extracellular matrix (dECM)‐based material provides low injection force and minimizes swelling, ensuring sustained volume. Combining CMC with supercritical fluid‐processed dECM enhances its effectiveness for long‐term wrinkle correction and sophisticated intradermal therapies.

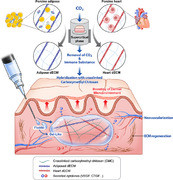

## 1 Introduction

Skin aging is a natural process that involves gradual changes in biomechanical function. The dermal extracellular matrix (ECM) deteriorates over time, accompanied by reduced cellular activity.^[^ ^1^ ^]^ As a result, the depletion of ECM components leads to a loss of skin tissue volume and a deterioration of structure, which manifests as decreased elasticity.^[^ ^2^ ^]^ Consequently, soft tissue augmentation products, like injectable fillers with less invasiveness have been developed to correct wrinkles and restore skin elasticity. These fillers must be durable and maintain stable injectability to effectively address the movement and pressure points in the soft tissues.^[^ ^3^ ^]^ Furthermore, current high‐viscosity hyaluronic acid (HA) fillers typically require the use of large‐gauge needles or cannulas for application, complicating the procedure and potentially triggering immune reactions in the skin and subcutaneous tissues.^[^ ^4^, ^5^ ^]^ Hence, developing biocompatible materials that can sustain their form and volume over extended periods, even after material degradation, remains critical. This need has led to the exploration of biomaterial‐based medical devices to ensure safety and exceptional volume and shape retention for reconstructing large‐volume defects. In this context, maintaining some degree of volume post‐degradation and facilitating the regeneration of vascularized adipose tissues are crucial.

Decellularized tissues have gained significant attention as promising biological components for scaffolds that effectively preserve their volume and support tissue regeneration.^[^ ^6^ ^]^ This process involves removing cellular components while preserving or minimizing the loss of tissue‐specific extracellular matrix (ECM) properties.^[^ ^7^, ^8^ ^]^ Furthermore, the decellularized ECM (dECM) contains an abundant supply of tissue‐specific growth factors and signaling molecules.^[^ ^9^ ^]^ Its properties, including 3D structure, porosity, and mechanical characteristics, resemble those of natural tissues, rendering dECM highly effective for filling voids and promoting tissue regeneration^[^ ^10^ ^]^ through enriched cytokine content, surpassing conventional approaches in physiological functionality. These characteristics have led to the widespread use of dECMs in various medical applications, such as accelerated skin healing in severe wounds,^[^ ^11^, ^12^, ^13^ ^]^ functional regeneration in myocardial infarction,^[^ ^14^ ^]^ and peri‐implant soft tissue regeneration.^[^ ^15^ ^]^ In addition, the development of composite materials by integrating dECM components has resulted in a range of commercial dECM‐based products. Nonetheless, most applications remain focused on broad areas such as regenerative medicine and wound care, with comparatively limited exploration of their potential as soft‐tissue volumizing fillers (Table S1, Supporting Information).

Despite their effectiveness in tissue regeneration, dECMs have limitations when used alone to achieve superior physical properties in soft‐tissue augmentation, especially as body fillers intended to replace large tissue volumes. The primary component of dECMs, collagen, typically exhibits a low storage modulus and elastic viscosity, and their physical characteristics are compromised by rapid degradation within the body.^[^ ^16^, ^17^, ^18^ ^]^ To address these shortcomings, research has focused on developing composite materials by integrating dECM components. Commercially available injectable fillers now include a product that combines crosslinked HA with dECM powder derived from the skin;^[^ ^19^, ^20^ ^]^ however, they face several drawbacks, including needle clogging from particle presence during injection, decreased viscoelasticity caused by HA swelling, and reduced moldability from volume changes.^[^ ^21^, ^22^ ^]^

In this study, we combined decellularized adipose and heart tissues processed via supercritical fluid technology with a chitosan‐based injectable filler, known for its superior physical properties, to enhance the restoration capabilities of the dermal microenvironment in an injectable formulation. Various studies have explored the combination of dECM and chitosan for different applications, demonstrating their potential in creating scaffold‐type materials for such as nerve tissue engineering,^[^ ^23^ ^]^ antibacterial agents,^[^ ^24^ ^]^ and wound healing.^[^ ^25^, ^26^ ^]^ However, it is evident that while these composites have been investigated for a range of uses, none have utilized the supercritical fluid process (scCO_2_‐EtOH), nor have any examined the application of these materials as dermal tissue volumizing fillers. scCO_2_‐EtOH, which utilizes carbon dioxide and ethanol at ≈300 bar, removes immunogenicity without the need for surfactants and retains ≈1.5–5‐fold more cytokines involved in vascular and adipose tissue induction compared with that in conventional methods while preserving over 80% of the bioactive substances found in natural tissue.^[^ ^27^, ^28^, ^29^ ^]^ We synthesized dECM‐based hybrid fillers by creating a double‐crosslinked hydrogel that integrated PEG‐crosslinked carboxymethyl chitosan (CMC) with a composite dECM enriched with adipogenic and angiogenic factors. Another innovation of our work is the development of a stable injectable hydrogel based on dual‐crosslinking technology, which responds to in vivo conditions, expecting low, stable injection forces, enabling smooth administration, while its temperature‐ and body‐fluid‐sensitive properties allow it to gel upon injection, providing high mechanical strength and molding capabilities within the body. This approach offers several advantages, including reduced pain and immune response due to minimal tissue disruption during injection.

We hypothesized that combining these two dECM composites with CMC could revolutionize reconstructive therapies by enhancing their biocompatibility, stability, and regenerative potential. This approach is expected to improve clinical outcomes and patient satisfaction. To confirm these benefits, we analyzed the physical and chemical properties of the material, including its bioactive components, assessed its viscoelasticity and injection force, and validated its efficacy through vascularized tissue regeneration in a UVB‐induced photoaging mouse model.

## 2 Results and Discussion

### 2.1 Development and Application of Dual‐Crosslinked Injectable Fillers with dECM for Enhanced Vascularization and Tissue Regeneration

**Scheme 1:**
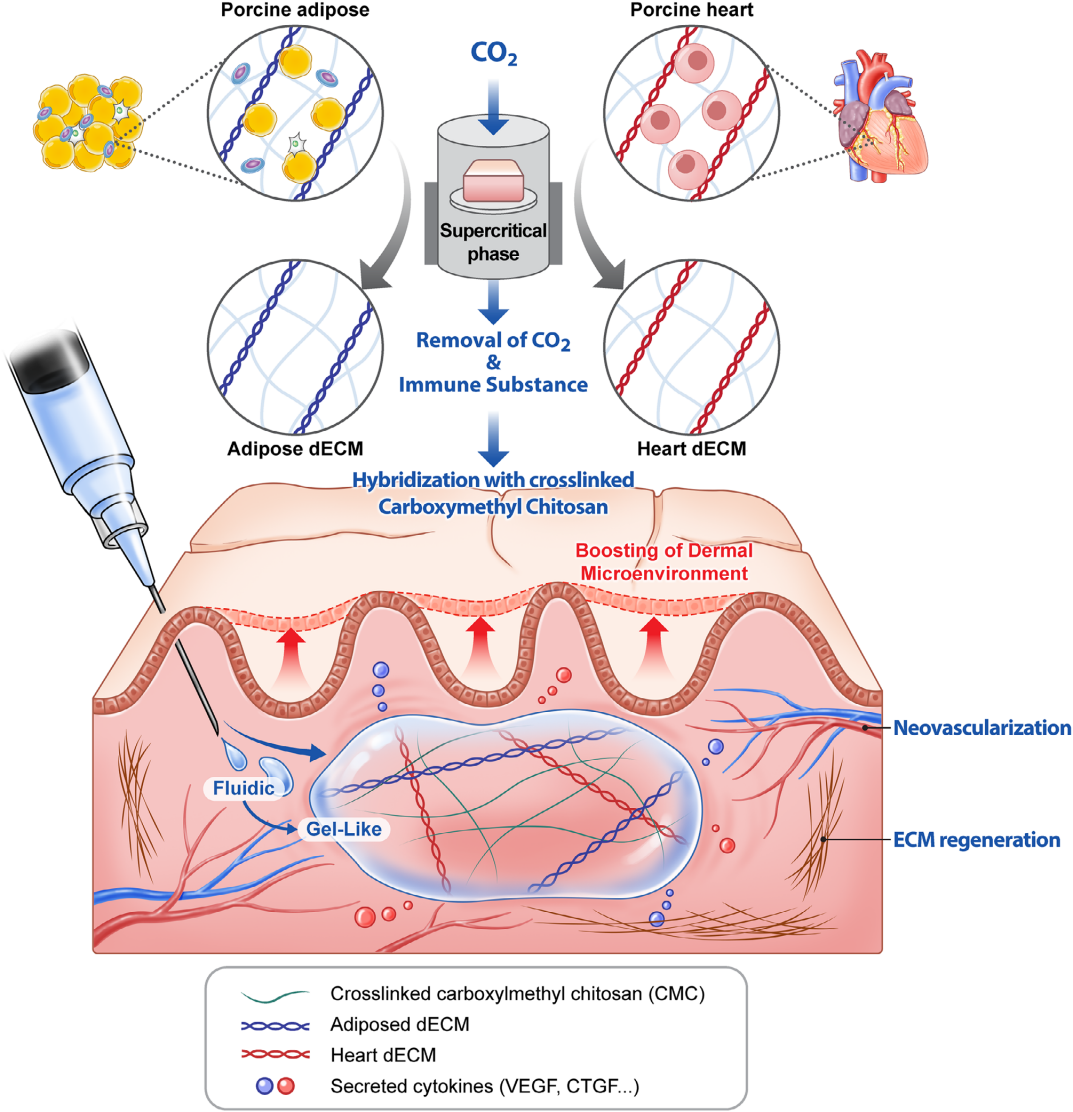
Overall fabrication of the dECM‐based multifunctional dermal filler: A) Schematic illustration of the decellularization process of porcine adipose and porcine heart tissue using supercritical CO_2_ system and hybridization process of dermal filler comprising carboxylmethyl chitosan (CMC) and dECM components. B) Application of the injectable filler for tissue boosting with regeneration via adipogenic and angiogenic factors from dECM multicomponents.

**Scheme** **1** presents a concise overview of the technology for developing dual‐crosslinked injectable materials incorporating decellularized adipose tissue (adECM) and decellularized heart tissue (hdECM) with CMC, designed to enhance vascularization and facilitate extensive tissue regeneration.

First, decellularized porcine tissue was successfully obtained using the scCO_2_‐EtOH process. This detergent‐free method eliminates nucleic acids and phospholipids from cells while preserving a high concentration of water‐soluble cytokines and structural components. Subsequently, an injectable dECM‐based hybrid filler was formulated through the hybridization of CMC with dECM components produced from the scCO_2_‐EtOH process. Briefly, decellularized tissue was rehydrated and mixed with CMC, which is prepared by a covalent reaction between PEGDE and CMC. These multicomponent fillers helped achieve superior injectability and moldability through a two‐step double crosslinking process. In the first phase, covalent crosslinking of CMC with PEGDE formed a fundamental matrix with liquid‐like properties, facilitating easy injection. In the second phase, the filler undergoes in situ crosslinking within the body owing to the presence of hybridized dECM components. This composite material functions as a dual‐functional filler, providing immediate volumetric support through molding and enhancing vascularized tissue regeneration. These hybrid dECM‐based fillers are promising candidates for anti‐aging dermal treatments (Scheme 1). They may help enhance the dermal connective tissue matrix within the dermal microenvironment by promoting angiogenesis driven by the abundant secretion of angiogenic and adipogenic factors from the injected dECM segments.

### 2.2 Characterization of Decellularized Porcine Tissue

**Figure 1:**
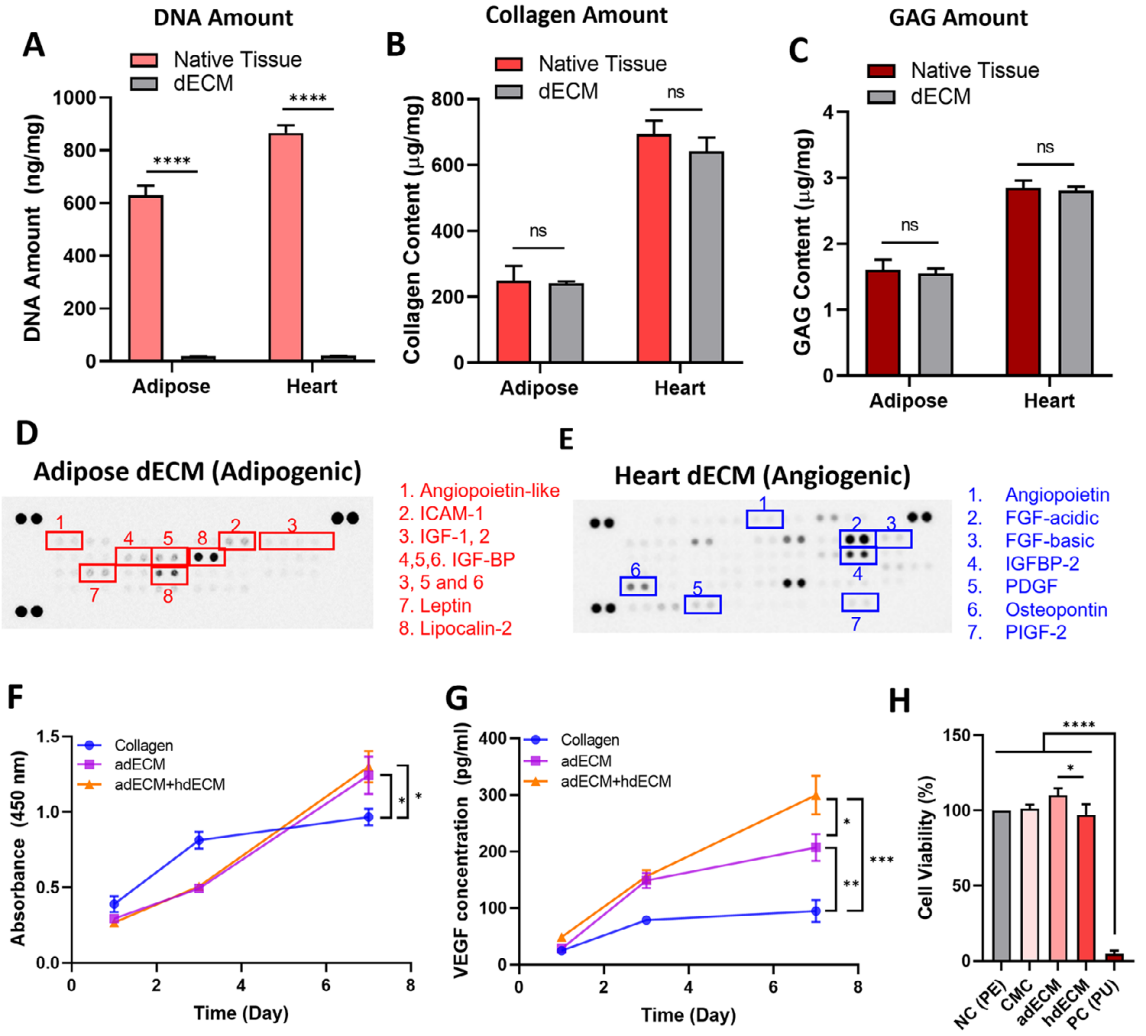
Characterization of adipose and heart dECM components: Quantitative analysis of residual A) DNA contents (*n* = 3, **** *p* < 0.0001) B) Collagen (*n* = 3) and C) glycosaminoglycan (GAG) (*n* = 3). Cytokine array showing adipogenic factors released from D) adipose and E) heart dECMs. F) Quantitative analysis of cell proliferation in preadipocytes of collagen, adECM, and hybrid dECM over time (*n* = 3, * *p* < 0.01). G) Quantitative analysis of released VEGF from pre‐adipocytes after culturing those cells with collagen, adECM, and hybrid dECM over time (*n* = 3, * *p* < 0.01, ** *p* < 0.001, *** *p* < 0.0001). H) Cell viability of L929 on the samples after 1 day of culturing (*n* = 3) (**** *p* < 0.0001, * *p* < 0.01).

The hybrid dECM derived from both adipose and heart tissues was used to promote vascularized adipose tissue formation. Initial quantitative analyses were performed to assess the effectiveness of immunogenicity removal using the scCO_2_‐EtOH process. Residual DNA levels in adipose dECM and heart dECM were measured at 19.93 ± 0.61 and 21 ± 1.59 ng mg^−1^, respectively, indicating a removal efficiency of 96.83% and 97.57% compared with that of native tissues (**Figure** **1**A). Xenogeneic biological tissues are frequently employed in regenerative medicine, with porcine tissues demonstrating over 70% cross‐reactivity with human tissues, making them suitable for human transplantation.^[^ ^30^ ^]^ Nonetheless, removing immune‐related components, including genetic material, is crucial for successful transplantation. The scCO_2_‐EtOH process, known for its ability to extract active ingredients by manipulating their polarity and thermodynamic properties, offers excellent biocompatibility for the production of decellularized materials. Less than 50 ng dsDNA mg^−1^ reportedly helps eliminate immune responses associated with xenografts,^[^ ^31^ ^]^ indicating that our material is suitable as an injectable augmentation. A key advantage of decellularized materials is their rich content of complex bioactive components, which are crucial for tissue regeneration. We analyzed the active ingredients in adipose dECM (adECM) and heart dECM (hdECM) prepared using scCO_2_‐EtOH. Quantitative assessments revealed collagen concentrations of 241.57 ± 5.36 and 643.22 ± 40.66 µg mg^−1^, respectively, preserving 96.73% and 92.65% of the native tissue levels (Figure 1B). The adECM and hdECM exhibited glycosaminoglycan (GAG) concentrations of 1.55 ± 0.07 and 2.8 ± 0.05 µg mg^−1^, respectively, retaining 96.73% and 98.48% of the native tissue content (Figure 1C). Cytokine profiling via dot blot analysis confirmed that the adipose tissue is rich in adipogenic cytokines, such as insulin growth factors, lipocalin‐2, and leptin (Figure 1D), whereas heart tissue is abundant in angiogenic factors, including angiopoietin and platelet‐derived growth factor (Figure 1E).

These findings align closely with those of previous studies that have shown that adipogenic cytokines play a crucial role in tissue regeneration and that angiogenic factors are vital for vascular development.^[^ ^32^ ^]^ Kim et al. compared heart dECM produced using detergent‐based methods and the scCO_2_‐EtOH process^[^ ^31^ ^]^ and reported that the dECM generated using the supercritical fluid technique contained up to sixfold more active ingredients than that in detergent‐processed dECM. This increase in active components translates to enhanced efficiency in promoting angiogenesis and tissue regeneration. Kim et al. also demonstrated that combining adipogenic and angiogenic factors effectively enhances both angiogenesis and the differentiation of progenitor cells. This leads to the development of vascularized adipose tissue that matures and maintains its functions, such as lipid accumulation, over time.^[^ ^32^ ^]^ Given these findings, our material manufacturing approach stands out for its superior multifunctionality. This ensures higher biocompatibility, as well as facilitates simultaneous angiogenesis and differentiation through the combined use of adipose dECM and heart dECM. The effects of adipose and hybrid dECM on preadipocyte behavior were compared with those of collagen. While pre‐adipocyte adhesion and proliferation were higher in the collagen group up to day 3, the dECM group showed a higher proliferation rate after day 3 (Figure 1F). By day 7, dECM‐treated preadipocytes exhibited over a 28% increase in viable cells compared with that in the collagen‐treated cells. No significant difference in cell proliferation was observed between the adipose and hybrid dECM groups.

The potential of adipose and hybrid dECM to stimulate vascular endothelial growth factor (VEGF) secretion from pre‐adipocytes was evaluated (Figure 1G). On day 7, both types of dECM significantly upregulated VEGF signaling by 118.67% for adipose dECM and 216.32% for hybrid dECM relative to collagen. Furthermore, hybrid dECM, which combines adipose and heart dECM, demonstrated 44.65% greater biological activity compared with that of adipose dECM alone. The dECM contains diverse cytokines depending on their origin, which influence cellular behaviors such as proliferation and differentiation. Boer et al. explored the impact of insulin‐like growth factor‐1 (IGF‐1) on human mesenchymal stromal cells and showed that IGF‐1 enhances proliferation and regulates gene expression.^[^ ^33^ ^]^ Additionally, Kim et al. found that the angiogenic factors in the heart dECM promote angiogenesis by increasing vWF+ expression in human umbilical vein endothelial cells, suggesting that this process facilitates angiogenesis in vivo and supports the regeneration of mature tissue.^[^ ^34^ ^]^

Biological safety assessments were performed in compliance with ISO 10 993 standards (Figure 1H). None of the raw materials demonstrated cytotoxicity in L929 cells. CMC was crosslinked with PEGDE, a crosslinking agent with lower toxicity than 1,4‐Butanediol diglycidyl ether (BDDE),^[^ ^35^ ^]^ and biocompatibility was further confirmed by eliminating residual crosslinking agents through dialysis. The dECM was manufactured using a supercritical fluid technique that avoided the use of harmful detergents, thereby maintaining the nontoxic nature of the material. Irritation tests were also conducted (Figure S1, Supporting Information). Intradermal injections of the extract did not induce any adverse reactions such as irritation, erythema, or swelling in any of the test groups. These results confirm the exceptional biological safety of this material, rendering it highly suitable for use in injectable medical devices.

### 2.3 In Vitro Physical Properties of dECM‐Based Hybrid Fillers

**Figure 2:**
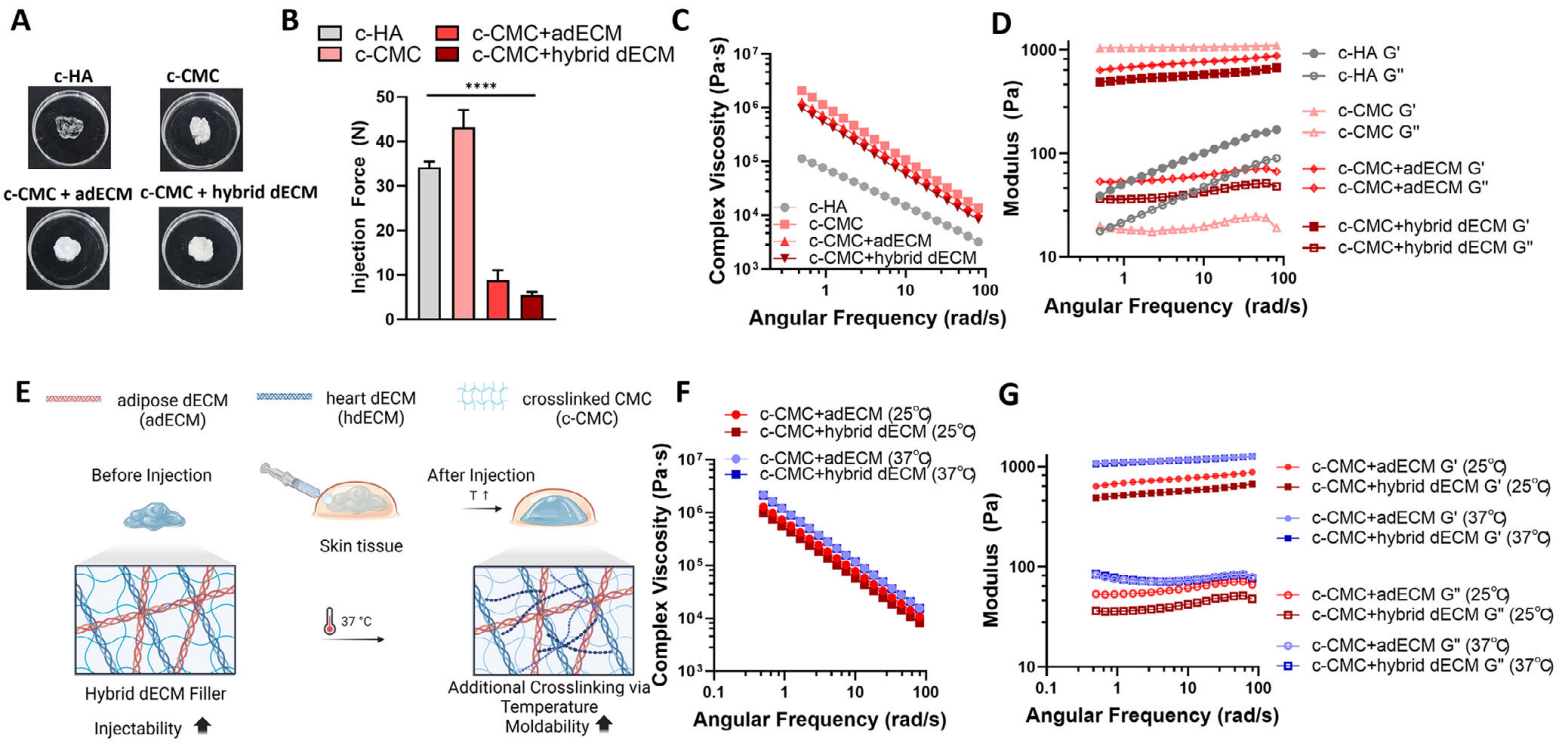
Physical characterization of dECM multicomponent dermal filler compared with HA and CMC as control groups: A) Photos of fillers including HA, CMC, and dECM hybrid fillers (CMC + adECM, CMC + hybrid dECM). B) Injection force of fillers (*n* = 50, **** *p* < 0.0001). C) Complex viscosity of fillers as a function of angular frequency. D) Storage and loss modulus of fillers as a function of angular frequency. E) Schematic illustration of dECM hybrid filler via temperature crosslinking. F) Change in viscosity of fillers as a function of temperature. G) Storage and loss modulus of fillers as a function of temperature.

Macroscopically, HA fillers appeared transparent, whereas intrinsic CMC fillers and those hybridized with CMC and dECM components were opaque (**Figure** **2**A). The filler used for augmentation exhibited significant viscoelasticity, with injection force influenced by its physical characteristics. In injectable materials, excessive crosslinking is often employed to enhance moldability, but this can lead to increased injection forces. Such elevated forces can cause discomfort during the procedure and may give rise to complications, including a higher risk of damaging blood vessels.^[^ ^36^, ^37^ ^]^ Specifically, injury to ophthalmic and retinal arteries during facial filler injections can lead to irreversible adverse events, or filler particles entering a vessel could trigger an inflammatory response, potentially obstructing blood flow.^[^ ^38^, ^39^ ^]^ In contrast, the combination of CMC and dECM in this study demonstrates much lower injection forces than HA, while still allowing for improved crosslinking. The CMC+dECM filler exhibited considerable viscoelasticity and adaptability within the body, as dECM underwent additional crosslinking in conjunction with PEG‐crosslinked CMC. Nevertheless, a low and stable injection force was maintained. The injection forces for CMC + adECM and CMC + hybrid dECM measured at 8.81 ± 2.21 and 5.46 ± 0.73 N, respectively, representing reductions of 74.19% and 84.00% relative to the 34.16 ± 1.31 N required for HA (Figure 2B). The relatively high injection force observed for HA can be attributed to its particulate form. The reduction in injection force, along with the temperature‐sensitive characteristics of dECM, results in a formulation that combines low injection force with high viscoelasticity once inside the body.

Particles comparable in size to the diameter of the 26‐gauge needle used for injection (≈130 µm) likely result in significant frictional forces during the injection process, thereby contributing to a higher measured injection force. Conversely, dECM‐based fillers contained significantly less CMC than those composed solely of CMC. Additionally, since the dECM was fabricated in a non‐crosslinked, non‐particulate form, it passed through the needle with a much lower injection force relative to other fillers. Despite its lower injection force relative to HA, CMC + dECM maintained impressive viscoelasticity. At a frequency of 0.5 rad s^−1^, the complex viscosities for HA, CMC + adECM, and CMC + hybrid dECM are 1.13 × 10^5^ Pa s, 12.77 × 10^5^ Pa s, and 9.77 × 10^5^ Pa s, respectively, with CMC + dECM demonstrating superior viscoelastic properties compared with that of HA (Figure 2C). The dECM hydrogel within the CMC + dECM filler formed a nanofibrous network in the body, enhancing its physical properties. The storage modulus values of each group showed the same tendency as the elastic viscosity (Figure 2D). Figure 2E illustrates the temperature‐sensitive physical properties of dECM, which enables low injection force and high viscoelasticity in the body. Figure 2F,G shows the elastic viscosity and modulus before and after incubation at 37 °C. The storage modulus of CMC + adECM and CMC + hybrid dECM increased from 636.59 and 487.3 Pa to 1080.03 and 1063.06 Pa, respectively, reflecting increases of 69.65% and 118.15% compared with those before incubation. Ozturk et al. reported that dECM derived from various sources showed more than a 200% improvement in physical properties at 37 °C compared with that at 25 °C.^[^ ^40^ ^]^

### 2.4 In Vivo Physical Properties of dECM‐Based Hybrid Fillers

**Figure 3:**
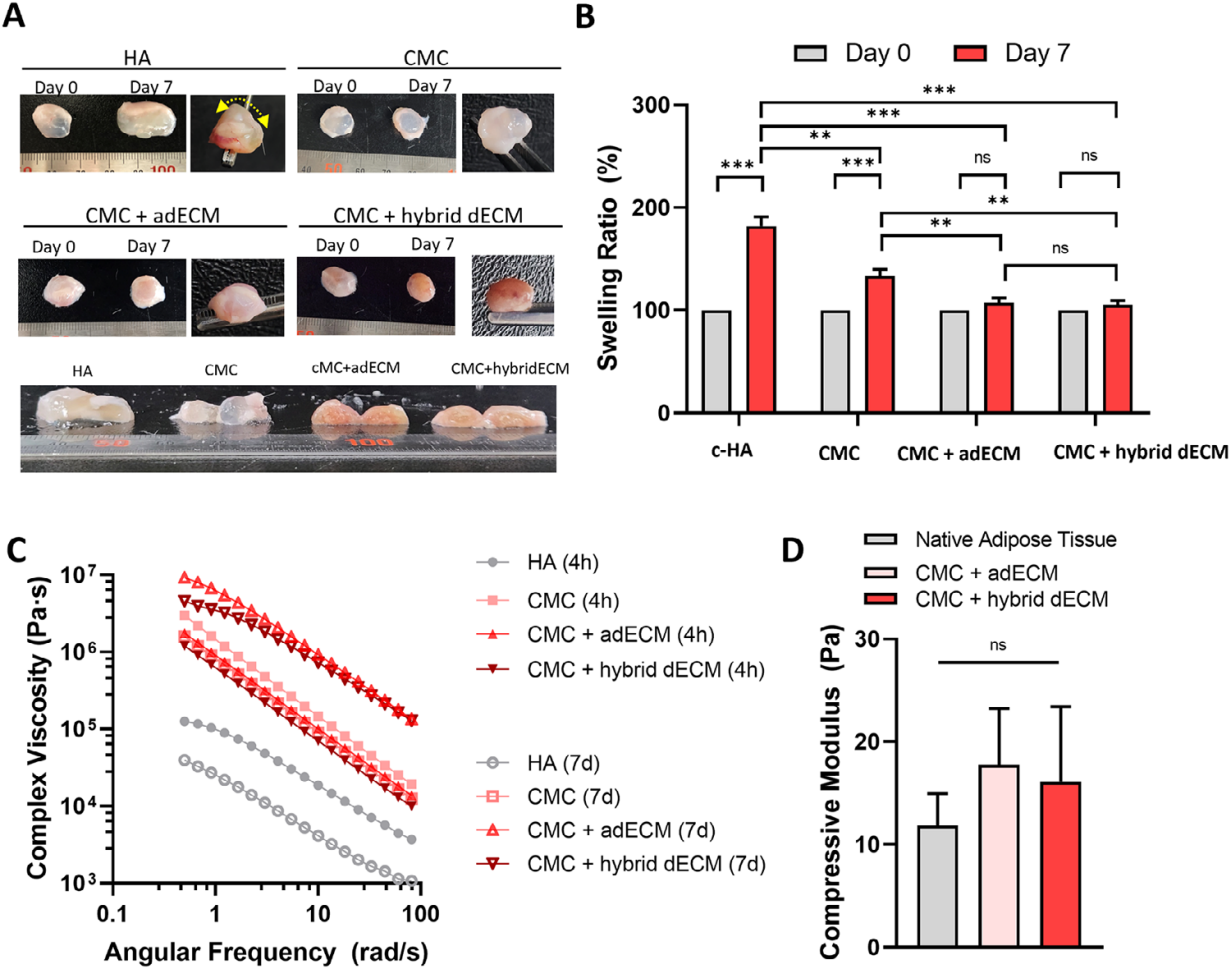
In vivo behavior of the hydrogels: A) Appearance of materials on day 0 (4 h post‐injection) and day 7 post‐subcutaneous injection. B) Swelling ratio of the materials. (*n* = 3, *t*‐test, ns: *p* > 0.05, *p ≤ 0.05, **p ≤ 0.01, ***p ≤ 0.001) C) Complex viscosity of the materials 4 h and 7 days post‐subcutaneous injection. D) Compressive modulus of CMC+ adECM and CMC + hybrid dECM 7 days post‐subcutaneous injection compared with that of the native adipose tissue (*n* = 3).

The physical properties of the dECM hydrogel were assessed through in situ crosslinking studies, as described in the literature.^[^ ^41^ ^]^ **Figure** **3**A shows the in vivo appearance of the samples. Immediately after injection, HA appeared spherical but swelled and became more fluid over seven days. Although CMC exhibited less swelling than HA, it underwent some expansion. Movie S1 (Supporting Information) demonstrates the physical properties when compression was applied to samples harvested 7 days post‐subcutaneous injection. In the dECM‐containing groups, minimal swelling was observed even on day 7 post‐injection, with consistent maintenance of shape and size. In addition, especially, a healthy light‐pink coloration with visible vascular structures from CMC + hybrid dECM, suggesting the formation of vascularized tissue near the filler surface. This observation provides intuitive visual evidence supporting our hypothesis that hybrid dECM materials play a role in enhancing vascularity while simultaneously promoting tissue integrity at the filling site as the material degrades, thereby improving the integration with the surrounding host tissue.

Hydrogels are hydrophilic; they absorb water from the body and maintain their volume. Although crosslinked HA is a biopolymer with significant water absorption, this characteristic makes it challenging to predict its shape and volume after injection. This unpredictability can result in undesirable side effects, such as dripping, owing to their relatively weak physical properties. Conversely, the CMC + dECM hydrogel minimizes excessive swelling, effectively maintains shape and volume over time, and enhances physical properties through improved engraftment and moldability via host–tissue interactions. Figure 3B presents the swelling measurements of the materials in the body. Initially, the volume of all hydrogels was set to 100%. HA swelled to 182 ± 8.88%, CMC to 133.66 ± 6.11%, CMC + adECM to 107.33 ± 4.72%, and CMC + hybrid dECM to 105.33 ± 4.16%. CMC exhibits a relatively lower water absorption rate than that of HA owing to its inherent material characteristics. Akbuga et al. studied the swelling ratios of HA and chitosan and discovered that increased HA content relative to chitosan resulted in a higher swelling ratio.^[^ ^42^ ^]^ Similarly, Zhang et al. reported that as the molecular weight of PEG increased, its water absorption capacity and swelling ratio also increased.^[^ ^43^ ^]^ In this study, PEGDE (Mn 500) was utilized to control the swelling ratio. Consequently, the CMC hydrogels exhibited a milder swelling response than that of crosslinked HA. Furthermore, in the group where dECM was combined with CMC, the initial volume remained nearly constant, with minimal changes observed. This stability is attributed to the ability of DCM to balance swelling owing to its composition resembling that of native tissue as well as the enhanced moldability provided by additional crosslinking in the body.

Following the subcutaneous injection and molding of the CMC + dECM hydrogel, an additional crosslinking step involving dECM was performed. Figure 3C and Figure S3 (Supporting Information) illustrate the changes in viscoelasticity before and after injection, respectively, demonstrating an enhancement in the material properties within a physiological environment. Figure 3C illustrates the viscoelastic properties of the materials after in vivo injection. On day 7, the complex viscosity of HA and CMC measured at 3.96 × 10^4^ and 1.58 × 10^6^ Pa s, respectively, representing reductions of 68.56% and 46.69% relative to their values on day 0 post‐injection. Conversely, the two dECM‐containing groups showed improved physical properties on day 7 compared with that on day 0. Specifically, the complex viscosities of CMC + adECM and CMC + hybrid dECM on day 7 were 9.25 × 10^6^ and 4.6 × 10^6^ Pa s, reflecting increases of 525.7% and 382.18%, respectively, relative to those on day 0. This enhancement is attributed to the in situ crosslinking of dECM within the body and its interaction with the host tissue, which helps to reflect the inherent physical properties of the tissue. Specifically, the storage moduli of CMC + adECM and CMC+hybrid dECM increased by 37.73% and 23.16%, respectively, post‐injection (Figure S3, Supporting Information). These findings indicate that the material retains optimal physical properties after injection with a low injection force, thereby reducing the potential side effects associated with higher injection forces and offering superior performance as a filler for soft tissue augmentation.

Closely matching the mechanical properties of biomaterials used in tissue regeneration with those of the surrounding tissues is crucial. Discrepancies in the mechanical properties can lead to immune responses or unnatural results. Our materials may help maintain their efficacy by promoting host tissue integration and exhibit stable physical properties. Figure 3D presents the comparison of the compressive modulus of native adipose tissue with that of the CMC + dECM hydrogels. According to existing literature, Young's modulus of human adipose tissue ranges from 17–23 kPa.^[^ ^44^ ^]^ Thus, our findings suggest that the injected material possesses physical properties similar to those of the surrounding host tissue.

The objective of the development of the composite material with CMC and dECM in this study is to make it gradually degrade over time after injection, inducing vascularized tissue at the treatment site while maintaining a stable volume. One of the key components, CMC, is a polysaccharide derived from natural polymers, and chitosan is known to degrade through lysozyme, an enzyme present in the body.^[^ ^45^ ^]^ Figure S4 (Supporting Information) illustrates that CMC undergoes biodegradation in a concentration‐dependent manner when exposed to lysozyme. Even in low‐concentration lysozyme solutions (0.2 mg ml^−1^), ≈70% of the injected CMC's molecular weight remains intact after 30 days, suggesting a relatively high stability against enzymatic degradation. Considering that lysozyme in the body exists at a concentration of 7–13 µg mL^−1^,^[^ ^46^ ^]^ significantly lower than the 0.2 mg ml^−1^ used in this experiment, it is expected that CMC will degrade gradually over a long period, ensuring long‐term biostability after injection.

### 2.5 Enhanced Wrinkle Reduction and Stability of CMC and dECM Fillers in Photoaged Skin

**Figure 4:**
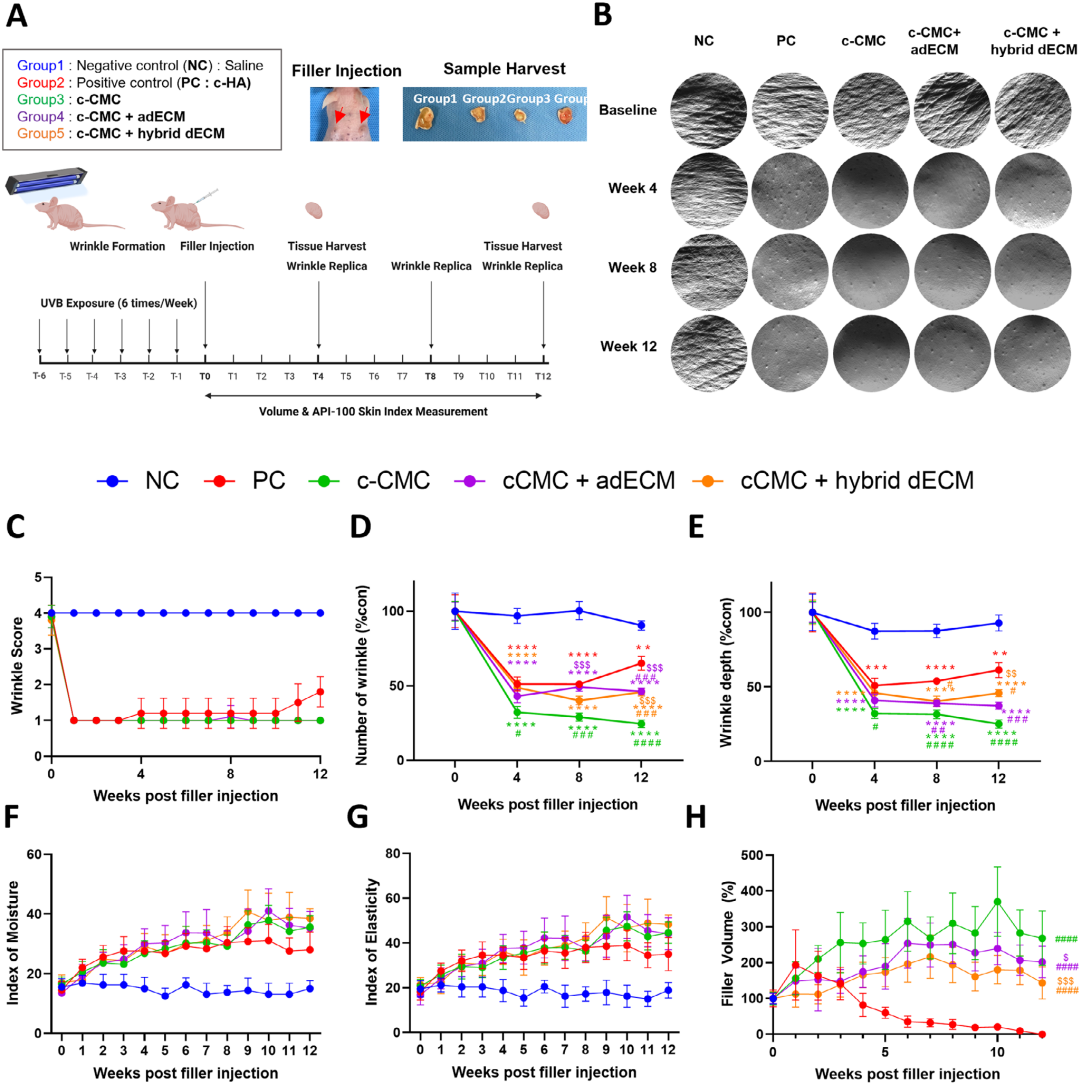
In vivo experimental setup and evaluation of the characterization of the wrinkles and filler volume change over time: A) Schematic timeline explaining the photoaging process and filler injection and wrinkle observation over time. B) Gross view of wrinkles analyzed using skin replica. C) Wrinkle score graph post‐filler injection. D) Number of wrinkles graph (**p < 0.01, ***p < 0.001, ****p < 0.0001, compared with NC, and HA). E) Wrinkle depth graph post‐filler injection (**p < 0.01, ***p < 0.001, ****p < 0.0001, compared with NC, # *p* < 0.01, ###*p* < 0.001, compared with PC, $$ *p *< 0.001, compared with CMC). F) Index of moisture graph post‐filler injection. G) Index of elasticity graph post‐filler injection. H) Filler volume change post‐filler injection, #### *p* < 0.0001, compared with PC, $ *p* < 0.01, $$$ *p* < 0.001, compared with CMC).

Using a photoaging model, we induced wrinkle formation and assessed the tissue augmentation performance of the dECM‐based hybrid fillers compared with that in fillers based on saline only (NC), ‐HA as a positive control (PC), and CMC through visual observation of wrinkles over time and silicone skin replica analysis (Scheme 1, timeline in **Figure** **4**A). With an increase in UVB radiation exposure, the severity of wrinkles on the skin of mice progressively worsened, confirming the effectiveness of the UVB irradiation model for simulating photoaging‐induced skin wrinkles, which is in line with previous findings.^[^ ^2^, ^47^, ^48^, ^49^ ^]^ (Figure S2, Supporting Information). The wrinkle evaluation was conducted 4, 8, and 12 weeks before and after the filler injection. Measurements such as the total wrinkle area and mean wrinkle depth were obtained, and statistical analysis was performed to assess the extent of wrinkles across the groups at each time point. Representative images of replica analysis at 4, 8, and 12 weeks after the filler injection are presented in Figure 4B.

These images illustrate that pre‐injection, numerous deep wrinkles were present. Post‐injection, improvements were observed across all groups compared with that in the NC group, with the CMC filler showing slightly more improvement than that in the PC group. According to API‐100 measurements, the negative control group maintained a consistent wrinkle score of 4 points throughout the assessment period. In the filler‐injected groups, the scores remained at 1 until the third week. Subsequently, in the PC group injected with HA fillers, the scores showed a slight increase from the fourth week onward, reaching 2 points by week 12. Conversely, the groups injected with CMC consistently maintained a score of 1 point from the first week through week 12 across all groups. These results suggest that, from the perspective of wrinkle score evaluation, the CMC and CMC + dECM fillers demonstrate superior effectiveness compared with that of PC (Figure 4C).

Based on the wrinkle index results, the graphs present the number of wrinkles counted for each group and the measured depth of the wrinkles (Figure 4D,E). The graphs illustrate the percentage change relative to the pre‐injection value, set at 100% for each group. These results suggest that NC exhibited minimal changes in both the number and depth of wrinkles over time. In contrast, all groups that received filler injections showed reductions of ≈≥50% in wrinkles 4 weeks post‐injection. Notably, the group injected with CMC alone demonstrated a reduction of over 70% in the number and depth of wrinkles at 4 weeks. Groups 4 (CMC + adECM) and 5 (CMC + hybrid dECM) also showed superior effects compared with those of the PC group. Furthermore, 12 weeks post‐injection, the PC group exhibited increased wrinkles, whereas the CMC group showed reduced wrinkle numbers and depths. These findings indicate that the CMC filler has significantly better wrinkle‐reducing effects than that of PC. Additionally, the evaluation of wrinkle severity at 12 weeks post‐injection revealed that the effectiveness of the HA filler diminished over time, whereas that of the CMC filler demonstrated increasing effectiveness. Moreover, the group injected with the CMC filler alone showed better results than the groups in which the CMC filler was combined with dECM. These results suggest that CMC plays a significant role in wrinkle improvement. The lower proportion of CMC in the dECM‐hybridized fillers compared with that in the CMC‐only group explains this outcome. Nevertheless, the fillers containing dECM showed significantly better wrinkle correction than those containing HA at 8 and 12 weeks post‐injection. This indicates that dECM‐containing fillers are more effective for wrinkle correction than those with HA.

The moisture levels in the filler injection groups increased compared with those in the NC group, with a gradual increase in the PC group that plateaued from week 4 (Figure 4F). The elasticity values exhibited a trend similar to that of the moisture levels (Figure 4G). The PC group also tended to reach saturation over time, which was consistent with the wrinkle area outcomes observed for the other fillers. Overall, a slight increase in wrinkle area was observed from week 8 after injection compared with that just before injection. This suggests that the CMC and CMC + dECM fillers may offer enhanced stability within the body, maintaining consistent skin moisture and elasticity. Conversely, HA gradually lost its volume over time, ultimately diminishing its ability to provide moisture and elasticity.

The volumes of the fillers were measured weekly using calipers at two sites in each of the five groups (Figure 4H). Results showed that the volume of HA (PC) steadily decreased from week 1 and rapidly diminished from week 3 onward. Conversely, CMC fillers exhibited a continuous increase in volume until the third week, and this increased volume was maintained until week 12. Additionally, in the group in which adipose tissue was decellularized (Group 4, CMC + adECM), although a slight reduction in volume compared with that in the c‐ group was observed, the volume was sustained from week 5 onward. Similarly, CMC + adECM and CMC + hybrid dECM also showed variations in filler volume but demonstrated good volume retention over time. These findings indicate that chitosan fillers outperform conventional HA fillers in terms of volumetric sustainability.

### 2.6 Biological Performance of Fillers in Dermal Matrix

#### 2.6.1 Reduced Immune Response and Enhanced VEGF Level in Dermal Matrix Treated with dECM‐Based Fillers

**Figure 5:**
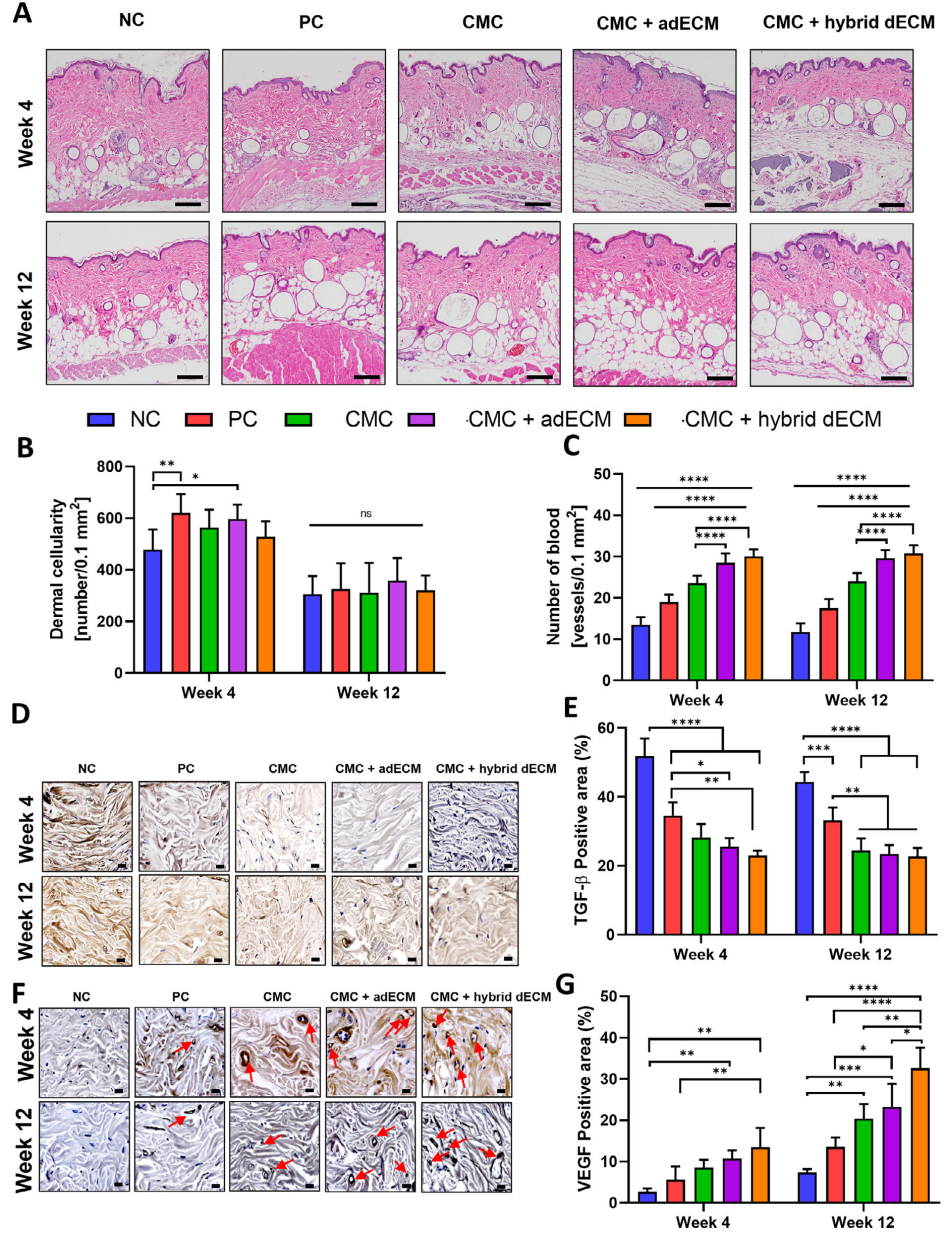
Histological and IHC images at weeks 4 and 12 post‐filler injection: A) Observation of dermis thickness using H&E staining at weeks 4 and 12 post‐filler injection. B) Quantified dermal cellularity graph post‐filler injection (* *p *< 0.05, ** *p* < 0.01). C) Quantified vascularity graph post‐filler injection (**** *p *< 0.0001). D) IHC stained images of TGF‐β post‐filler injection at weeks 4 and 12 (Scale bar: 20 µm). E) Calculated TGF‐β positive area graph post‐filler injection * *p* < 0.05, ** *p* < 0.01, *** *p* < 0.001, **** *p* < 0.0001). F) IHC stained images of VEGF post‐filler injection at weeks 4 and 12 (Scale bar: 20 µm). G) Calculated VEGF positive area graph post‐filler injection * *p* < 0.05, ** *p *< 0.01, *** *p* < 0.001, **** *p* < 0.0001).

Although photoaging and natural skin aging involve different mechanisms, observing recovery characteristics of dermal tissue damage in photoaged models can effectively validate the potential of dECM‐based fillers as viable therapeutic materials for tissue regeneration.^[^ ^50^, ^51^, ^52^ ^]^ Our dECM‐based fillers, composed of multiple dECM components with significantly low immunogenicity achieved through scCO_2_‐EtOH processing,^[^ ^53^ ^]^ are expected to demonstrate significantly reduced cellularity, primarily driven by the hybrid filler group. We assessed cellularity within the tissues through hematoxylin and eosin (H&E) staining, quantifying the number of inflammatory cells per unit area to gauge the extent of the inflammatory response elicited by the inserted fillers. Representative images of H&E staining are shown in **Figure** **5**A.

Analysis revealed that at 4 weeks, the PC group injected with the HA filler showed an increase in inflammatory cell count, indicating an inflammatory response (Figure 5B). The group injected with CMC also exhibited increased cellularity compared with that of the NC group, although to a lesser extent than that in the PC group. Conversely, the CMC + hybrid dECM group showed cellularity levels similar to those of the NC group (no statistical difference). However, the CMC + adECM group showed a slightly increased level of cellularity compared with that of the NC group. These findings suggest that CMC‐based fillers induce less inflammation than HA fillers, and injecting CMC + hybrid dECM results in even lower inflammatory responses than that induced by injecting CMC + adECM. Additionally, at 12 weeks post‐injection, we observed no significant differences in cellularity between the groups. This suggests that despite differences in filler volume retention, filler efficacy did not significantly differ after the long‐term observation period. The observed increase in blood vessels indicated a promotion of vascularity, with all filler groups showing significant efficacy by weeks 4 and 12 (Figure 5C). Notably, both the CMC + adECM and CMC + hybrid dECM filler groups exhibited substantially increased vascularity than that in the NC, PC, and CMC groups. This indicates that dECMs play a role in enhancing vascularity. These results are consistent with those of earlier in vitro studies demonstrating the impact of dECM on vascular development. The dECM, containing various cytokines depending on its origin, affects cellular processes such as proliferation and differentiation. The increased VEGF secretion observed with dECM suggests that it contributes to elevated VEGF levels in the photoaged dermis, thus enhancing vascular formation when the fillers are hybridized with additional dECM sourced from porcine heart tissue.

After injecting fillers, we assessed the inflammatory response by staining for transforming growth factor‐beta (TGF‐β), which mediates tissue inflammation and can lead to fibrosis, excessive ECM accumulation, and tissue stiffening, thereby impairing tissue function. Notably, during photoaging, TGF‐β levels increase and become activated, driving the overproduction of matrix metalloproteinases and pro‐inflammatory cytokines.^[^ ^54^ ^]^ This sustained neutrophil infiltration leads to continuous collagen degradation and the formation of abnormal elastic fibers, which together contribute to ECM breakdown (Figure 5D). Quantitative analysis of staining intensity using ImageJ software showed that at week 4, the PC group exhibited ≈20% reduced inflammation compared with that in the NC group (Figure 5E). Similarly, the CMC filler group also showed reduced inflammation; however, the difference compared with that in the PC group was not statistically significant. CMC + adECM and CMC + hybrid dECM both showed significantly lower inflammation than that of PC, indicating a trend toward a reduced inflammatory response. This trend was most evident in the CMC + hybrid dECM at week 4, which exhibited the lowest level of inflammatory response.

VEGF plays a crucial role in tissue regeneration following filler injection. It promotes the proliferation of endothelial cells within the blood vessels, inducing the formation of new vessels to supply nutrients and oxygen to damaged tissues, thereby accelerating the regeneration process. Additionally, VEGF stimulates the proliferation and migration of various cells, including fibroblasts, aiding cell movement to wound sites and tissue regeneration. By modulating inflammatory responses, VEGF ensures that the initial inflammation positively influences tissue regeneration. These properties highlight VEGF as a key factor promoting tissue regeneration post‐filler injection. From the immunostained images of VEGF (Figure 5F), at week 4, the PC group showed an approximately twofold increase in VEGF expression compared with that in the NC group (Figure 5G). The CMC group demonstrated over 1.6‐fold higher expression than that in the PC group.

Furthermore, both the CMC + adECM and CMC + hybrid dECM groups exhibited higher VEGF expression than the PC group. These results were statistically significant for the CMC + hybrid dECM (** *p *< 0.01), demonstrating the highest impact on VEGF expression. Additionally, at week 12, the overall VEGF expression decreased slightly compared with that at week 4; however, filler injections in groups 4 and 5 still resulted in significantly higher VEGF expression compared with that in the other groups. Collectively, at weeks 4 and 12, the CMC + hybrid dECM showed markedly elevated VEGF expression, indicating its high potential for promoting neovascularization and regeneration in human skin tissue. Notably, this effect was over twice as pronounced compared with that in the PC group. Additionally, CMC + hybrid dECM showed significantly higher levels of VEGF expression than that of CMC + adECM at week 12, indicating that heart dECM provided more angiogenic factors than adipose dECM to evenly bring about vascularized dermal tissue.

#### 2.6.2 Enhanced Mature ECM Formation in Dermis Treated with dECM‐Based Fillers

**Figure 6:**
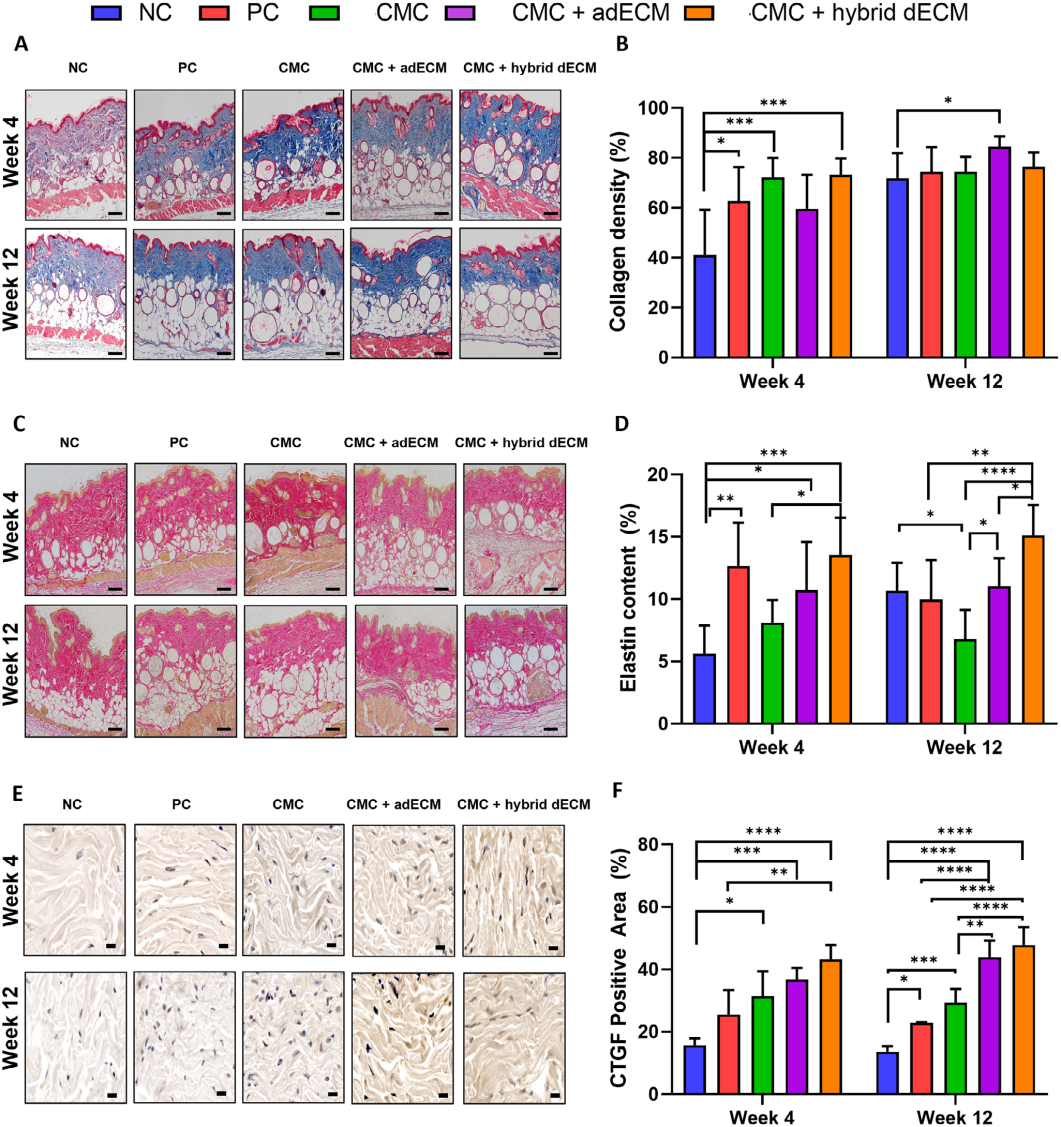
Histological and IHC images at weeks 4 and 12 post‐filler injection: A) Observation of collagen distribution using MT staining at weeks 4 and 8 post‐filler injection (Scale bar: 100 µm). B) Quantified collagen density graph post‐filler injection (* *p *< 0.05, *** *p *< 0.001). C) Elastic fiber formation in the dermis observed from images stained with Verhoeff–Van Gieson at weeks 4 and 12 post‐filler injection (Scale bar: 100 µm). D) Quantified elastin fiber graph post‐filler injection * *p *< 0.05, ** *p *< 0.01, *** *p* < 0.001, **** *p* < 0.0001). E) IHC stained images of CTGF post‐filler injection at weeks 4 and 12 (Scale bar: 20 µm). F) Calculated CTGF positive area graph post‐filler injection (* *p* < 0.05, ** *p *< 0.01, *** *p *< 0.001, **** *p* < 0.0001).

Masson's trichrome (MT) staining, used to stain collagen, a key ECM component,^[^ ^55^ ^]^ indicated effective tissue and skin regeneration through increased collagen staining after filler injection. Representative images of MT staining results for each group are shown in **Figure** **6**A. At week 4, the PC group exhibited a significant increase of over 50% in collagen compared with that in the NC group. Specifically, CMC exhibited a greater increase in collagen compared with that in the PC group (Figure 6B). Similarly, the CMC + hybrid dECM demonstrated higher collagen synthesis than that in the NC group. Statistically, groups 3 and 5 demonstrated a significantly higher collagen increase than that in the PC group, whereas no significant difference was observed between PC and CMC + hybrid dECM. Consistent with the H&E staining results, by week 12, the differences in collagen density between the fillers were not substantial, with collagen density remaining higher than that of the NC group but showing no significant variation among the filler groups. CMC + adECM exhibited a significantly higher collagen density than that in the NC group at week 12, indicating its enhanced collagen synthesis capacity compared with that of the other fillers.

Analysis of Verhoeff–Van Gieson‐stained images (VVG) revealed a notable increase in elastin levels in the CMC + dECM groups (both groups 4 and 5) compared with those in the CMC and NC groups (Figure 6C). At week 4, the elastin content in the CMC + adECM group was not significantly different from that in the CMC group. In contrast, the ratio of elastin formation in the CMC + hybrid dECM group was substantially higher than in the CMC group (Figure 6D). By week 12, the CMC + hybrid dECM group exhibited the highest concentration of quantified elastin fibers compared with both the CMC and CMC + adECM groups, as well as the other control groups. This suggests that the hybridized dECM, incorporating both adECM and hdECM, significantly accelerated the deposition of connective tissue components, likely owing to the enhanced secretion of cytokine factors from the two dECM types.

Connective tissue growth factor (CTGF) plays a crucial role in tissue regeneration by promoting cell proliferation and differentiation and enhancing the synthesis of ECM proteins such as collagen and elastin, which help maintain tissue structure.^[^ ^56^ ^]^ Moreover, it stimulates vascular neogenesis to supply nutrients and oxygen to damaged tissues and regulates inflammation to support tissue regeneration.^[^ ^57^ ^]^ Therefore, an increase in CTGF expression indicates that fillers enhance tissue regeneration and the synthesis of ECM proteins (Figure 6E). At week 4, the PC group showed an ≈1.5‐fold increase in CTGF expression compared with that in the NC group (Figure 6F). CMC + adECM exhibited an approximately twofold increase, with a similar level of CTGF expression as that in the PC group. CMC + hybrid dECM demonstrated the highest CTGF expression, ≈2.8‐fold higher than that in the NC and CMC groups, and this increase was statistically significant. Furthermore, upon evaluating CTGF expression at week 12 compared with week 4, the CMC + adECM and CMC + hybrid dECM groups showed substantially higher expression levels than those in the PC and CMC groups. Group 5 exhibited the highest CTGF expression among all groups at week 12, and notably, the CMC + hybrid dECM showed higher CTGF expression than the CMC + adECM.

Collectively, from week 4 to week 12, CTGF expression increased more prominently in the heart dECM group, indicating that the effects of the filler on tissue regeneration and ECM component synthesis are most effective in this group. Kim et al. demonstrated that angiogenic factors, such as VEGF, in the cardiac dECM promote the formation of new blood vessels and positively impact the key cells involved in tissue regeneration.^[^ ^35^ ^]^ They found that enhanced vascularization led to more mature tissue development. Our findings align with this, revealing that the hybrid dECM stimulates a prolonged release of VEGF, which accelerates collagen synthesis and improves elastin deposition, thereby supporting the ECM characteristics of mature skin.

## 3 Conclusion

In conclusion, our findings significantly advance the field of biomaterials for tissue repair and medical devices by introducing multifunctionality that enhances large‐scale volume retention. We achieve this by optimizing mature tissue regeneration efficiency while providing exceptional properties such as high viscoelasticity, moldability, and the capacity to support vascularized tissue formation. The soft nature of dECM materials ensures a low injection force and promotes additional crosslinking effects within the body, resulting in stable moldability at the injection site with minimal lateral diffusion in the direction of skin tension after injection.

Additionally, while injectable fillers require high water absorption to lift the tissue matrix, traditional materials such as HA or CMC can cause excessive swelling over time, potentially leading to unwanted skin pressure and immune reactions. Our findings suggest that dECM‐based substitutes are more suitable for long‐term wrinkle correction because they maintain volume persistence with minimal adverse effects.

Furthermore, the hybridization of biocompatible CMC with decellularized materials processed using supercritical fluid technology holds significant promise for advancing soft tissue regenerative therapies. Notably, the addition of adECM and hdECM to these dermal fillers enhances their multifunctional properties, improving dermal matrix reprogramming by increasing connective tissue components and angiogenic cytokine factors, in comparison to traditional fillers based solely on HA or CMC. Therefore, the filler proposed in this study offers an elegant solution for skin care, serving as a sophisticated “biorevitalization” agent or a cutting‐edge alternative for advanced intradermal therapy. However, there are notable limitations in the current study. A key challenge is the difficulty in fully understanding the specific signaling pathways through which adipose and heart‐derived dECM materials influence the dermal microenvironment. Future research should focus on a more in‐depth investigation into the molecular mechanisms by which dECM interacts with the dermal environment. Additionally, further exploration of the combination with other bioactive materials or fine‐tuning of the dECM ratios with varying dECM concentrations and crosslinking levels could lead to more substantial advancements in tissue regeneration and volume retention. Furthermore, beyond small animal models like rats, larger animal experiments, such as those conducted with rabbits, are necessary to confirm the biocompatibility, longevity, stability, and efficacy of the materials. Ultimately, these efforts will be crucial in refining the preclinical protocol and ensuring the successful translation of this technology to clinical applications.

## 4 Experimental Section

### Materials

Phosphate‐buffered saline (PBS, 20×) was procured from T&I and used at a 1× dilution. Ethyl alcohol (anhydrous, 99.5%) was purchased from Daejung Chemicals and diluted to 70%, 50%, and 30% concentrations before use. Deoxyribonuclease I from bovine pancreas (DNase, ≥400 Kunits mg^−1^ protein), polyethylene glycol diglycidyl ether (PEGDE, average molecular weight 500), acetic acid, pepsin from porcine gastric mucosa (≥400 units mg^−1^ protein), papain from papaya latex (≥10 units mg^−1^ protein), cysteine, ethylenediaminetetraacetic acid (EDTA), and dimethylmethylene blue (DMB) were obtained from Sigma‐Aldrich. Sterile distilled water (DW) was obtained from JW Pharmaceuticals. CMC was procured from HMC+ (Germany). Sodium hydroxide (NaOH) and isopropanol (IPA, 99.5%) were obtained from Duksan Pure Chemicals Co., Ltd. Hydrochloric acid (HCl) was acquired from Samchun Chemicals, and Triton X‐100 was obtained from Biosesang.

### Decellularization of Porcine Adipose and Heart Tissues

For the adipose dECM, non‐adipose tissues were excised from the adipose tissue and cut into sections ≤3 mm. Blood was completely removed by soaking the tissues in PBS and mechanically stirring overnight. Lipid components in the adipose tissue were eliminated by stirring for 48 h using isopropanol. The samples were subsequently washed with DW for 48 h and freeze‐dried for 72 h. The dried tissues were immersed in 99.5% anhydrous ethanol and incubated overnight. Ethanol was removed, and decellularization was conducted using a supercritical carbon dioxide autoclave (Ilshin Autoclave Co., Ltd., Daejeon, Korea) at 300 bar, 35 °C, 200 rpm for 6 h with scCO₂‐EtOH (supercritical CO₂ + 99.5% anhydrous ethanol). The decellularized adipose tissue was rehydrated in the following sequence: 99.5%, 70%, 50%, 30% ethanol, and PBS. The rehydrated tissue was stirred with a DNase solution (deoxyribonuclease + PBS) for 48 h. The adipose dECM was lyophilized for 72 h and washed with DW for 48 h. The freeze‐dried adipose dECM was ground into a fine powder using a cryomiller (FreezerMill SPEX Sample Prep, Metuchen, NJ, USA).

For the heart dECM, the pericardium was removed, isolating only the endocardium and myocardium. A heparin solution (50000 U/500 mL) was used to remove all blood clots in the endocardium, which was then sliced to <3 mm in size. The tissue was soaked in PBS and manually stirred to remove all blood. Following 3 days of lyophilization, dried heart tissue was placed in 99.5% anhydrous ethanol and incubated overnight. The subsequent steps followed the same procedure as that of adipose dECM, from the supercritical treatment to the powdering process.

### Fabrication of Composite Filler with CMC

CMC was dissolved in sterile DW to create a 4% CMC solution. This solution was then combined with a 0.1 N sodium hydroxide solution at a 10:1 ratio using a digital rotator and mixed overnight. To crosslink the solution, 1.5% PEGDE was added, and the mixture was incubated at 60 °C for 4 h. PEGDE was removed by immersing the crosslinked CMC solution in PBS and washing for 24 h with a magnetic stirrer. The final product was sterilized by autoclaving at 121 °C for 15 min.

### Preparation of adECM and hdECM Hydrogels

adECM and hdECM (40 mg) were dissolved in 1 mL of 0.1 N HCl at 4 °C. Subsequently, 0.5 N NaOH was added to neutralize the pH. The dECM hydrogel, exhibiting uniform properties, was filtered through a 5‐µm filter. For the hybrid dECM‐based fillers, CMC was combined with a decellularized extracellular matrix (dECM) at a 4:6 volume ratio. In the CMC+adECM formulation, only adipose‐derived dECM was used. In the CMC+hybrid dECM filler, a blend of adipose and heart dECM was mixed at a 7:3 ratio.

### Characterization of Hybrid dECM‐Based Fillers

Residual DNA in the decellularized tissue was quantified using the DNeasy Blood and Tissue Kit (Qiagen, USA). Tissue samples (25 mg) were collected, and DNA was extracted using tissue lysis buffer. The DNA content (ng DNA mg^−1^ tissue) was analyzed using a NanoDrop (Thermo Fisher, ND‐1000, Seoul, Korea). Collagen content was quantified using a Sircol Collagen Assay Kit (Biocolor Life Science, Northern Ireland, UK). Briefly, collagen was extracted using cold acid‐pepsin (pepsin concentration, 2 mg mL^−1^ in 0.5 m acetic acid) at 4 °C. Following treatment with the dye reagent, the optical density was measured at 555 nm using a microplate reader. Glycosaminoglycan (GAG) concentration was measured using a dimethylmethylene blue (DMMB) assay. adECM, hdECM, and native tissues were homogenized and digested in a papain reagent containing 100 µg mL^−1^ papain, 100 mm phosphate buffer, 10 mm cysteine, and 10 mm EDTA for 18 h at 60 °C. The samples were treated with a DMB dye solution, and the absorbance was measured at 530 nm using a microplate reader. Cytokines involved in adipogenesis and angiogenesis in adipose and heart dECMs were investigated using a microarray kit (Proteome Profiler Array; R&D Systems, Minneapolis, MN, USA). Native and decellularized tissue samples were treated with 1% (v/v) Triton X‐100 in PBS containing protease inhibitors and chopped for digestion. After homogenization, freezing, thawing, and centrifugation for 5 min to remove cell debris, supernatants containing cytokines were added to each array membrane on a shaker overnight at 4 °C. Following treatment with the detection antibody, the Chemi Reagent Mix was added to the membranes. The membranes were then exposed to light using an X‐ray imaging system (RAS‐3000; Fuji, Aichi, Japan).

### Physical Properties of Fillers – Rheological Behaviors and Injectable Force Measurement

The injection force of the fillers was assessed using a force tester machine (MCT‐2150, A&D Company, Japan) equipped with a 500 N load capacity. Samples were dispensed using a 26‐gauge needle operating at an injection speed of 30 mm min^−1^. Viscosity was evaluated using a rotational rheometer (MCR 302, Anton Paar, Korea) to monitor complex viscosity, storage modulus, and loss modulus at an angular frequency range from 0.5–200 rads at 25 °c.

### Physical Properties of Fillers – Temperature Sweep

To confirm rheological behavior and physiological temperatures, fillers were incubated at 37 °C overnight. Viscosity was then measured at an angular frequency range from 0.5–200 rads at 37 °C.

### In Vitro Cell Viability Tests

Mouse fibroblast (L929) cells were cultured on a 150 mm plate and incubated in Dulbecco's modified eagle medium (DMEM, Welgene, Korea) supplemented with 10% fetal bovine serum (FBS) and 1% penicillin‐streptomycin (PS), in a humidified incubator with 5% CO₂ at 37 °C for 24 h. Post stabilization, L929 cells were seeded in 96‐well plates at a density of 1 × 10⁴ cells well^−1^. Samples were eluted at 37 °C for 24 h by adding culture medium to 0.2 g mL^−1^. The 96‐well plates were treated with 100 µL well^−1^ of the eluate and were incubated for 48 h. Following eluate removal and washing with DPBS, a reagent containing WST‐8 (MediFab, Korea) and culture medium mixed in a 1:10 ratio was dispensed at 110 µL well^−1^. Cells were then incubated for 2 h, and 96‐well plates were analyzed using a microplate reader at a wavelength of 450 nm.

### In Vitro Bioactivity Tests

Human preadipocytes were derived from subcutaneous adipose tissue samples obtained from the abdomens of seven women donors aged 35–54 years, with approval from the Catholic University of Korea Institutional Review Board. Preadipocytes were cultured at 37 °C and 5% CO_2_ for seven days. The 2 wt.% dECM hydrogel was mixed with the culture medium in a 1:1 ratio and changed every two days. Cell proliferation was assessed using the WST‐8 Viability Assay Kit (Medifab). VEGF levels were measured by collecting samples every two days during media exchanges, and ELISA (Duoset, R&D Systems)was performed.

### In Vivo Tests Using a Photoaging Model

Fifty male SCH1 Hairless mice (six weeks old) were procured from Orient Bio (Seongnam‐si, Gyeonggi‐do, Republic of Korea). All animal procedures adhered to the guidelines of the Institutional Animal Care and Use Committee (approval number 2022‐0032). A one‐week acclimatization period was provided in separate cages.

A photoaging mouse model was established to evaluate the efficacy and durability of the fillers.^[^ ^47^ ^]^ Briefly, wrinkle formation was induced in five mice per group using an acrylic rack and irradiation with UVB six times weekly for eight weeks using a UV illuminator (UV‐3000, BoTeck, Korea) equipped with a UVB lamp (312 nm). The UVB dosage was incrementally increased over the 8‐week period, culminating in a total exposure of 69.6 minimal erythemal dose (MED), equivalent to 10440 mJ cm^−2^ (Figure S2, Supporting Information). The MED signified the minimum UV radiation dose necessary to induce erythema.^[^ ^48^ ^]^

Two days post‐induction, fillers were injected in five groups: saline (Negative control, NC), HA (positive control, PC), CMC, CMC+adECM, and CMC+hybrid dECM. The injections, whether saline or filler, were administered using a 1‐ml syringe (KOVAX‐SYRING, Koreavaccine, Republic of Korea) equipped with a 26‐gauge needle. Each injection consisted of 0.1 cc administered at two skin sites (left and right) per mouse. The study was conducted in two phases: 4‐ and 12‐week periods.

### Measurement of Injected Fillers and Wrinkle Analysis

Volumetric analysis of fillers and wrinkle assessment were performed weekly. Volume measurements were taken at two sites per group using a Caliper (Absolute AOS Digimatic, Mitutoyo, Japan), calculated using the formula: Volume (mm^3^) = Major axis (mm) × Minor axis (mm) × Minor axis (mm) × 0.5. For wrinkle assessment, an API‐100 device (Aram Huvis Co., Ltd., Seongnam‐si, Gyeonggi‐do, Republic of Korea), which made contact with the mouse skin and employed a camera to assess numerical wrinkle scores was used. These scores were averaged for each group to determine wrinkle measurement results. Wrinkle assessments were conducted before filler injection and 4 and 12 weeks post‐injection. Under respiratory anesthesia, a replica locator ring was fixed at the injection site, and a silicone mold of the skin was obtained using silicone from the Repliflo Cartridge Kit (CuDerm Co., Dallas, TX, USA). Wrinkle analysis was performed on these molds using a skin viscometer VL650 device (Courage+Khazaka Electronic, Cologne, Germany) at an incident angle of 35° to analyze shadow images under consistent lighting conditions. Statistical analyses were performed on metrics such as total wrinkle area and mean wrinkle depth to assess wrinkle severity across groups at various time points.

### Histological Observation

At 4 and 12 weeks post‐treatment, the mice were euthanized following ethical guidelines in accordance with the ARRIVE 2.0 recommendations. The euthanasia was performed by exposing the mice to a CO_2_ chamber for a minimum of 5 min to ensure a humane and stress‐free death. After confirmation of death through the absence of respiration and reflexes, skin tissue samples were collected. These samples were fixed in 4% paraformaldehyde at 4 °C for 24 h and subsequently embedded in paraffin for further histological analysis. Sections of the paraffin‐embedded tissues (4‐µm thick) were stained with H&E, MT, and VVG stains following standard protocols. Each stained tissue group was examined at 100× magnification using an optical microscope (Olympus BX53; Olympus Corp., Shinjuku, Japan). Quantification of inflammatory cell area ratios, vascularity from H&E‐stained images, collagen density ratio from MT‐stained areas, and elastic fiber density ratio from VVG‐stained areas was performed using ImageJ software. Areas stained with H&E were used for calculations.

### Immunohistochemistry (IHC) Staining

Immunohistochemical staining was performed on 4‐µm thick paraffin sections obtained from 4‐ and 12‐week samples. Following fixation in 4% paraformaldehyde, sections were blocked with a mixture of 5% goat and horse sera at room temperature for 1 h. Primary antibodies against TGF‐β (1:100, Abcam Ab16667), CTGF (1:100, Abcam, ab6992), and VEGF (1:500, Santacruz sc‐57496) were applied overnight at 4 °C, followed by incubation with appropriate secondary antibodies for 1 h at room temperature. Whole‐slide scanning was performed using a ZEISS Axio Scan.Z1 microscope (Zeiss Group, Oberkochen, Germany) at 100× magnification and analyzed by calculating mean values from captured images.

### Biodegradation Test of Carboxymethylchitosan

The degradation of carboxymethylchitosan (CMC) by lysozyme was analyzed. 2w/v% CMC was added to 4 mg mL^−1^ lysozyme solution and then incubated at 37 °C for 30 days. The molecular weight of the sample before and after lysozyme treatment was measured using GPC (Gel permeation chromatography, HLC‐8420, Tosoh)

### Statistical Analysis

Statistical analyses were performed using GraphPad Prism to assess the significance of comparisons throughout the study. For comparisons involving multiple groups, one‐way analysis of variance (ANOVA) followed by Tukey's multiple comparison test was performed, with significance levels denoted as *P < 0.05, **P < 0.01, ***P < 0.001, and ****P < 0.0001. For comparisons between two groups, a two‐sided *t*‐test was employed, and significance levels were indicated as *P < 0.05, **P < 0.01, ***P < 0.001, and ****P < 0.0001.

## Conflict of Interest

The authors declare no conflict of interest.

## Supporting information



Supporting Information

Supplemental Movie 1

## Data Availability

Data sharing is not applicable to this article as no new data were created or analyzed in this study.
