# Supplementary material for: Supercritical Fluid‐Processed Multifunctional Hybrid Decellularized Extracellular Matrix with Chitosan Hydrogel for Improving Photoaged Dermis Microenvironment
Source: Adv Healthc Mater. 2025 Mar 20;14(11):2403213. doi: 10.1002/adhm.202403213 (PMC12023828; doi:10.1002/adhm.202403213)
Supplement: Supplementary file 1 — Supporting Information [file ADHM-14-0-s001.docx]

Supporting Information

# Supercritical Fluid-Processed Multifunctional Hybrid Decellularized Extracellular Matrix with Chitosan Hydrogel for Improving Photoaged Dermis Microenvironment

Seol-Ha Jeong^1,2^†, Jae Jun Kang^3^†, Ki-Myo Kim^1^, Mi Hyun lee^1^, Misun Cha^3^, Su Hee Kim^3*^, Ji-Ung Park^1,2,*^

1 Department of Plastic and Reconstructive Surgery, Seoul National University Boramae Hospital, Seoul National University College of Medicine, 07061, Seoul, Republic of Korea

2 Bio-max Institute, Seoul National University, 08826, Seoul, Republic of Korea

3 R&D Center, Medifab Co. Ltd, 5 Gasan digital 1-ro, Geumcheon-gu, Seoul 08594, Republic of Korea

†These authors contributed equally as first authors to this work.

* Co-corresponding author: Su Hee Kim, [sweess@imedifab.com](mailto:sweess@imedifab.com), & Ji-Ung Park, [parkjiung@snu.ac.kr](mailto:parkjiung@snu.ac.kr)

Keywords: adipose dECM, carboxymethylchitosan hydrogel, decellularization, dermal fillers, heart dECM, photoaging, supercritical fluid processing

Table S1. Summary of products containing dECM materials for injectable filler applications

| **Injectable Filler Component** | **Type / Content** | **Applications** | **Manufacturing Company** |
| --- | --- | --- | --- |
| Porcine skin dECM | Matrix type 1cm x 5cm ~ 5cm x 10cm | Wound care | ACRO biomedical |
| Decellularized Dermal Particle, Collagen type Ⅰ | 1ml | Aesthetic Medicine | ACRO biomedical |
| Decellularized Porcine Organ | liver scaffold sheet / thickness : 0.1~2mm  kidney scaffold sheet / thickness : 1~3mm  brain scaffold sheet / thickness : 3~5mm  pancrea scaffold sheet / thickness : 1~2mm  heart scaffold sheet / thickness : 1~2mm  blood vessel scaffold / diameter : 3~5mm, length : 2~4mm  nerve scaffold / diameter : 1~2mm, length : 5 / 10 / 15 / 20mm  ureter scaffold / diameter : 3~5mm, length : 2cm | Regenerative Medical Study | ACRO biomedical |
| Human derived Acellular Dermal Matrix + HA | 1 ml | Wound Dressing | L&CBIO |
| Human Skin Acellular Dermal Matrix + crosslinked HA | Gel type / 1 ml | Regenerative Biomatrial | L&CBIO |
| Crosslinked Human Skin Acellular Dermal Matrix | Sheet type, Power type, Carving type | Regenerative Biomatrial | L&CBIO |
| **Porcine Adipose dECM, Heart dECM, crosslinked carboxymethylchitosan** | **Gel type** | **Therapeutic Soft tissue Volumizing Filler** | **This Study** |
|  |  |  |  |


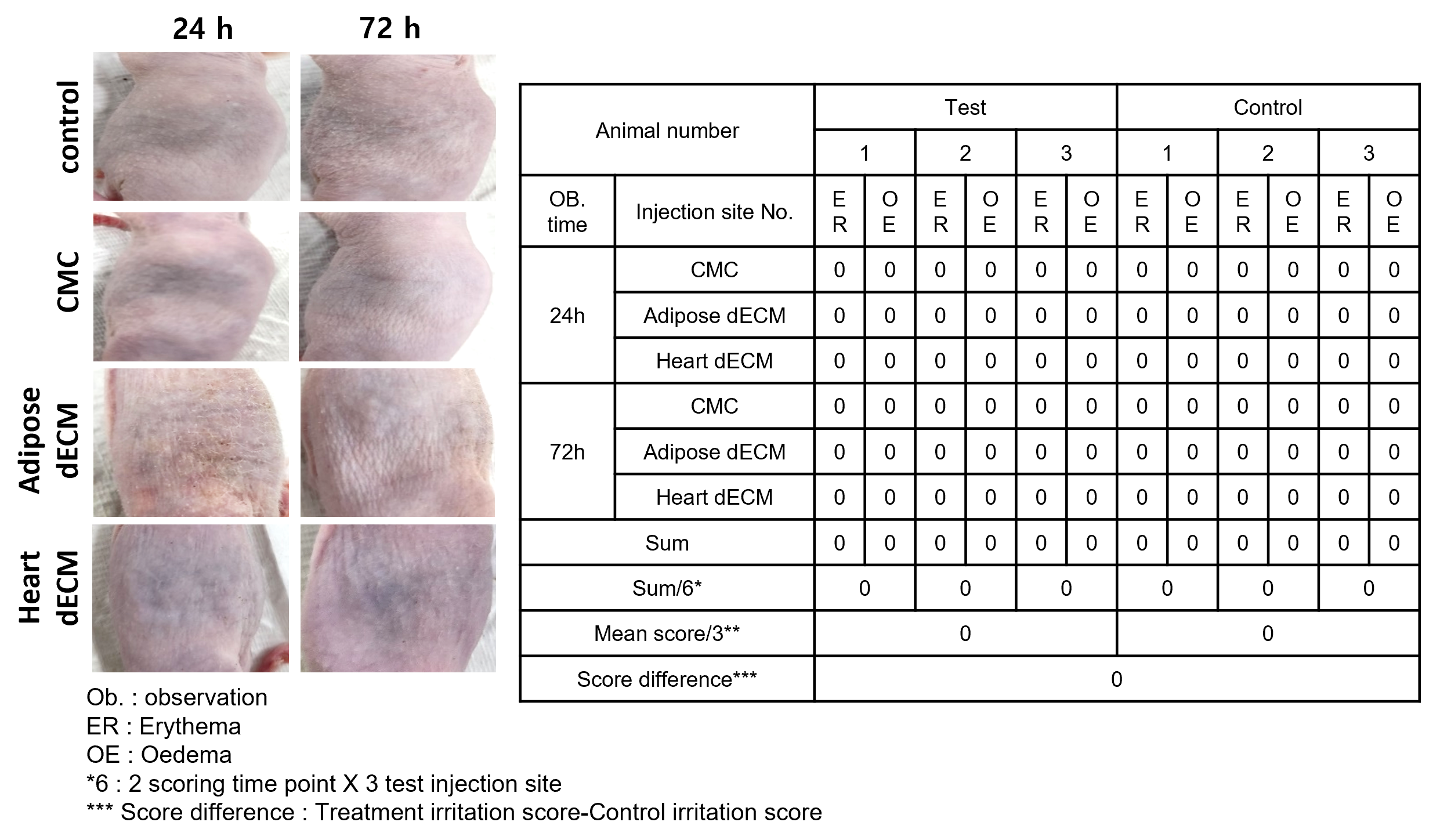


Figure S1. Irritability test after filler injection to the subcutaneous site of nude mice (intradermal reactivity).


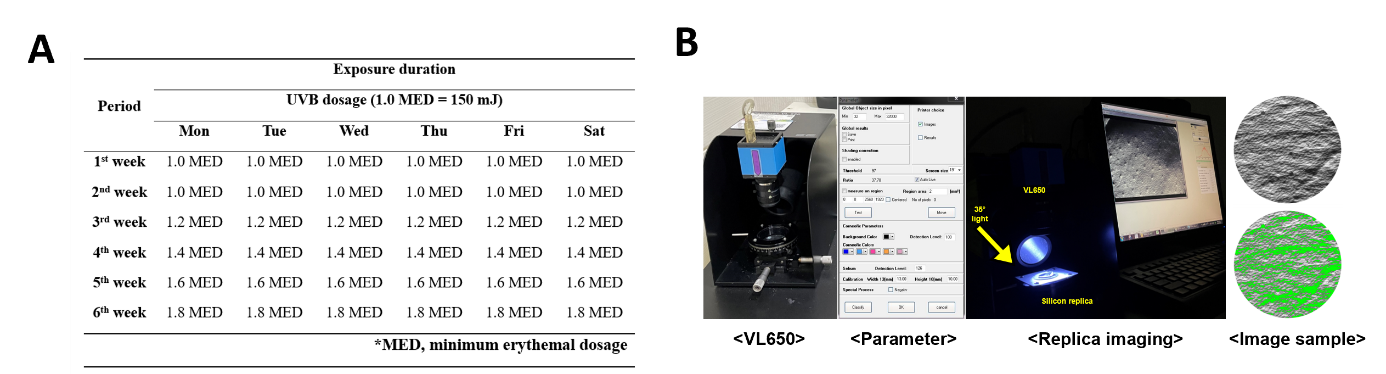


Figure S2. (A) Summary of UVB exposure duration. (B) Examples of measurements using the wrinkle analysis device (Skin Visiometer VL650).


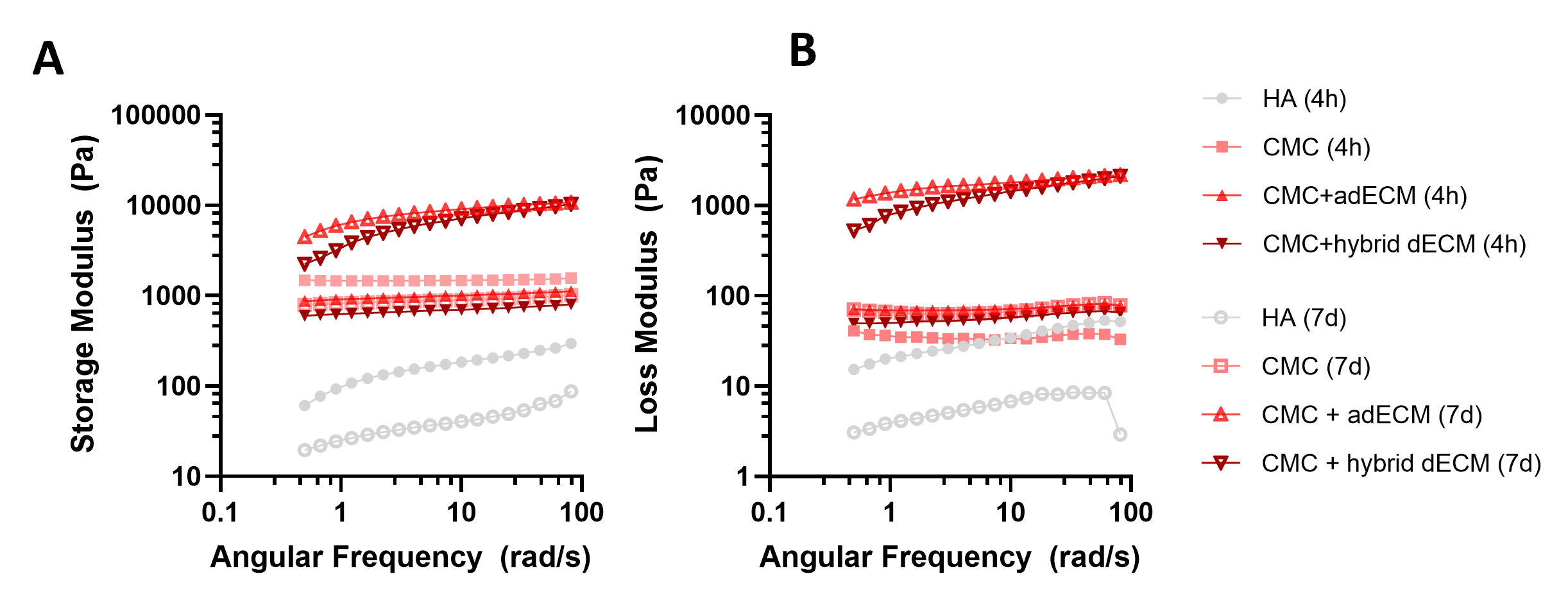


Figure S3. Physical properties of the samples under physiological conditions. (A) Storage and (B) loss moduli of the fillers.

Figure S4. *In vitro* degradation profile of CMC under lysozyme with different concentration (n=3).
